# Unraveling the Genetic Threads of History: mtDNA HVS-I Analysis Reveals the Ancient Past of the Aburra Valley

**DOI:** 10.3390/genes14112036

**Published:** 2023-11-02

**Authors:** Daniel Uricoechea Patiño, Andrew Collins, Oscar Julián Romero García, Gustavo Santos Vecino, Pablo Aristizábal Espinosa, Jaime Eduardo Bernal Villegas, Escilda Benavides Benitez, Saray Vergara Muñoz, Ignacio Briceño Balcázar

**Affiliations:** 1 Doctoral Program in Biosciences, Human Genetics Group, Faculty of Medicine, University of La Sabana, Chía 250001, Colombia; daniel.uricoechea@unisabana.edu.co; 2 Human Genetics & Genomic Medicine, Faculty of Medicine, University of Southampton, Southampton SO16 6YD, UK; a.r.collins@soton.ac.uk; 3 Genetics Group, National Institute of Legal Medicine and Forensic Sciences, Bogotá 110311, Colombia; 4 Department of Anthropology, Faculty of Social and Human Science, Universidad de Antioquia, Medellín 050010, Colombia; gsantosvecino@yahoo.es; 5 School for Advanced Studies in the Social Sciences, Americanists Society of Paris, 75006 Paris, France; pabloaristizabal888@hotmail.com; 6 Faculty of Medicine, University of Sinú, Cartagena de Indias 130011, Colombia; jebernal@gmail.com (J.E.B.V.); sarayvergara@unisinu.edu.co (S.V.M.)

**Keywords:** ancient DNA, pre-hispanic, mtDNA HVS-I, haplogroup, aburrá valley, antioquia, Colombia

## Abstract

This article presents a comprehensive genetic study focused on pre-Hispanic individuals who inhabited the Aburrá Valley in Antioquia, Colombia, between the tenth and seventeenth centuries AD. Employing a genetic approach, the study analyzed maternal lineages using DNA samples obtained from skeletal remains. The results illuminate a remarkable degree of biological diversity within these populations and provide insights into their genetic connections with other ancient and indigenous groups across the American continent. The findings strongly support the widely accepted hypothesis that the migration of the first American settlers occurred through Beringia, a land bridge connecting Siberia to North America during the last Ice Age. Subsequently, these early settlers journeyed southward, crossing the North American ice cap. Of particular note, the study unveils the presence of ancestral lineages from Asian populations, which played a pivotal role in populating the Americas. The implications of these results extend beyond delineating migratory routes and settlement patterns of ancient populations. They also enrich our understanding of the genetic diversity inherent in indigenous populations of the region. By revealing the genetic heritage of pre-Hispanic individuals from the Aburrá Valley, this study offers valuable insights into the history of human migration and settlement in the Americas. Furthermore, it enhances our comprehension of the intricate genetic tapestry that characterizes indigenous communities in the area.

## 1. Introduction

The Antioquia region has played a pivotal role in the pre-Hispanic history of South America, serving as a gateway for the initial human migrations to the continent and as a hub for the development of a sophisticated culture deeply rooted in the utilization of natural resources and advanced technologies [1]. Colombia boasts a multitude of archaeological sites that have enabled the tracking of the earliest human arrivals, dating back to approximately sixteen thousand four hundred (16,400) ± four hundred twenty (420) A.P. [2], as determined through radiocarbon dating of artifacts linked to ancient human activities. Among these sites, Pubenza, nestled in the lowlands of the middle-valley of the Magdalena River [3], and El Jordán in the municipality of Roncesvalles (Tolima), stand out as some of the oldest, alongside El Totumo, El Abra, Tibitó, Tequendama, and Nemocón. All are strategically situated in the Sabana de Bogotá within the Eastern Cordillera [4] (Table 1).

The earliest radiocarbon-dated evidence within the Antioquia Department is prominently concentrated in the Medellín/Porce River basin and the Medellín/Porce River high basin (Table 1) [5]. Early agricultural societies in the region exhibit distinct burial practices, including simple pit burials and cremations conducted in ceramic vessels. These burials, in some instances, feature accompanying vessels used as lids or funerary ornaments [6]. Two distinct funerary patterns have emerged within Colombia.

The first pattern corresponds to the Marrón Inciso ceramic style, spanning from the first century BC to the eighth century AD. The second pattern corresponds to the Ferrería ceramic style, ranging from the fourth century BC to the fourth century AD. The Marrón Inciso style extends throughout the Cauca mountain basin, while the Ferrería style is prominent in the central mountain range of Antioquia. Cerro El Volador, situated within the city of Medellín, has yielded Marrón Inciso-style burials, including an urn containing cremated human remains and a necklace bead resembling a praying mantis, linking ceramics to gold craftsmanship. The second funerary pattern, originating in Antioquia in the fourteenth century AD, is associated with the late ceramic style, spanning from the tenth century AD to the time of the Conquest. This pattern is characterized by burials in intricate and spacious funerary structures known as “pit tombs with a lateral chamber”, commonly found atop hills in cemeteries [7].

Cerro El Volador, for example, revealed twelve tombs featuring rectangular access shafts and expansive chambers of conical and elliptical configurations. These tombs, dated between the fifteenth and seventeenth centuries AD, and contained cremated human remains, indigenous pottery fragments, skeletal remains of cattle and horses, along with European artifacts. The architectural design of these burial chambers mirrors indigenous dwellings or huts. At La Colinita’s summit, three “side chamber well tombs” unveiled cremated and non-cremated human remains, spindle ruffles, gold nose-rings, tumbaga, vessels, and ceramic fragments. Another burial site atop Las Flores contained the skeletal remains of an adult individual, devoid of grave goods but adorned with engravings representing a hut. The “pit tombs with a lateral chamber” in the Aburrá Valley are intricate representations of indigenous dwellings, holding profound significance both in construction and cosmology [7] These are hills with privileged panoramic views, where accumulations of tombs or necropolises were discovered. It should be clarified that the tombs investigated in the three hills of the Aburrá Valley, where the city of Medellín is located, belong to this latter period.

Molecular biology, leveraging DNA analysis, has played a pivotal role in shedding light on hypotheses concerning the origins of human populations. DNA, the repository of hereditary information and essential protein-coding data, was first structurally elucidated in 1953 by Watson, Crick, and Franklin. Subsequently, Sanger’s sequencing method paved the way for reading and decoding genetic information. Recent advancements in data storage, analytical technologies, and informatics have catalyzed breakthroughs across multiple disciplines, including archaeology. Despite the inevitable degradation of DNA post-mortem, it can adhere to hydroxyapatite in bones, maintaining sufficient quality for reconstructing ancient genomes.

Mitochondrial DNA (mtDNA) analysis, particularly in tracing maternal lineages, has emerged as a powerful tool for unraveling population history and genetic ancestry. The exclusive maternal transmission of mtDNA enables the tracking of maternal lineages across generations, facilitating the reconstruction of ancient population movements and migrations.

The high copy-number of mtDNA within cells empowers the recovery and examination of genetic information from ancient remains, thereby aiding our comprehension of genetic diversity and population dynamics over time. Maternal lineages, a focal point of mtDNA analysis, are identified through the detection of characteristic mutations that serve as markers for distinct population groups. These findings have significantly contributed to the development of theories regarding the settlement of pre-Hispanic communities in Colombia.

Ancient DNA studies have sought to unravel the genetic structure of pre-Colombian populations and establish genetic links between historical and contemporary population groups. These investigations are instrumental in supporting models related to population replacements, migrations, and genetic admixtures. As of now, genetic ancestry data in the Aburrá Valley are scarce.

To gain a more comprehensive understanding of population dynamics, migration patterns, and genetic relationships within the region, future studies should consider incorporating complete nuclear and mitochondrial genome analysis. This advanced approach will enable a more detailed and precise assessment of the genetic history of the pre-Hispanic inhabitants in the Aburrá Valley; thereby, enriching our comprehension of the complex tapestry of human settlement and evolution in this pivotal region.

## 2. Materials and Methods

### 2.1. Sample Collection

This study was centered on the analysis of mitochondrial DNA HVS-I extracted from ten individuals originating from the Aburrá Valley in Antioquia. These individuals were associated with pit tombs characterized by lateral chambers. Gender determination was based on the anthropological examination of the skeletal remains.

**Figure 1:**
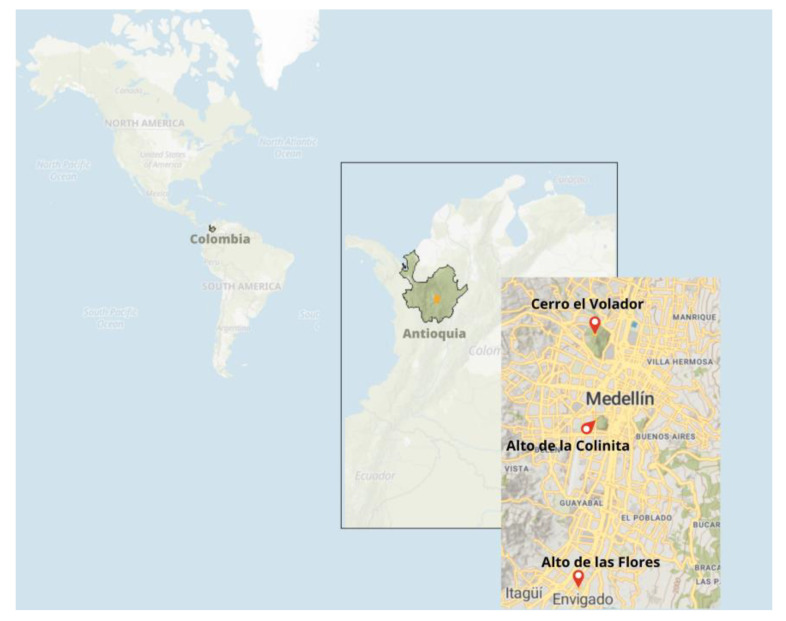
Geographic overview of excavation sites for pre-Hispanic human remains in the department of Antioquia, municipalities of Medellín, and Envigado.

We conducted research at three distinct archaeological sites: Cerro del Volador, which yielded one individual (bone); Alto de las Flores in Envigado, where we analyzed one individual (bone) in Medellín; and La Colinita, where we investigated eight individuals (molars) in Medellín (Figure 1). The samples selected for analysis date back to the late pre-Hispanic period and the contact period, specifically spanning from the eleventh to the eighteenth centuries AD [7].

### 2.2. Ancient DNA Extraction

The genetic analysis of individuals from bone remains was conducted at the Human Genetics Laboratory within the specialized area dedicated to archaic DNA procedures at the University of Sabana. Stringent authenticity criteria, designed to prevent contamination of ancient DNA as proposed by Cooper, Poinar, and Gilbert [8], were strictly adhered to during the entire process. Mitochondrial DNA sequences from the HVS-I region were detected through the polymerase chain reaction (PCR) amplification method. Subsequently, these sequences were aligned with the revised Cambridge Reference Sequence (rCRS) for detailed analysis [9].

To ensure the accuracy and reliability of the findings, the procedures were independently replicated by two researchers on separate occasions. Furthermore, a subset of the samples was sent to the Department of Genetic Epidemiology and Bioinformatics at the University of Southampton in England.

### 2.3. PCR Amplification and Sequencing

To determine the mitochondrial haplotypes of the individuals in this study, we focused on a 388 bp fragment of HVS-I, specifically nucleotide positions 16024–16408. For amplification, we employed four pairs of overlapping PCR primers that were specifically developed for pre-Hispanic American populations [10]: H15995: GCT AAG ATT CTA ATT TAA ACT ATT CT (26 bp); L16174: GGA TTG GGT TTT TAT GTA CTA C (22 bp); H16105: TGC CAG CCA CCA TGA ATA TTG TAC (24 bp); L16256: GCT TTG GAG TTG CAG TTG ATG TGT (24 bp); H16194: ATG CTT ACA AGC AAG TAC AGC AA (23 bp); L16360: GAG AAG GGA TTT GAC TGT AAT GTG (24 bp); H16261: CCT CAC CCA CTA GGA TAC CAA CAA (24 bp); L16429: CCT CAC CCA CTA GGA TAC CAA CAA (21 bp). For the amplification of the HVS-I in human bone and molar remains containing ancient DNA, a standardized process was employed. This process involved a final volume of 10 µL and PCR amplification utilizing a hot start Polymerase and dNTPs. The resulting PCR product was verified using a 2% agarose gel, and the most suitable bands were selected for Sanger sequencing. Stringent alignment and analysis was performed using the QIAGEN CLC Genomics Workbench 20.0.4 program. Variants were independently and repeatedly identified by three investigators; each was assigned a GenBank accession number within the range BankIt 2736055: OR478541–OR478622. Haplogroups and haplotypes were determined using the HaploGrep2 program. Variations with a quality score exceeding 60% present in at least three duplicate samples were documented to ensure the reliability of our findings.

### 2.4. Statistical Analyses

To conduct individual analyses, we employed specific haplotypes and constructed phylogenetic trees using unique polymorphisms for each haplogroup. We determined common evolutionary relationships through several approaches, including neighbor-joining algorithms, UPGMA, direct networks using the NETWORK 10 program, and the HaploGrep 2 program [11].

Our study also encompassed intra- and inter-population analyses, performed with the Arlequin 3.5 program. We calculated various indices to discern population trends, and mean pairwise *F_ST_* genetic distances were computed between populations based on their mitochondrial haplotype frequencies.

In this analysis, we considered twenty present-day populations from Colombia and seventy-seven pre-Hispanic American populations. These were categorized into eight groups based on previous research. We obtained distance matrices and visualized them using the MDS ALSCAL algorithm within the SPSS program. This method provides a balanced approach, addressing concerns regarding false positives and statistical power, as opposed to relying solely on phylogenetic trees [12,13,14,15].

## 3. Results

In our study, we identified a total of ten individuals, collectively representing the four founding haplogroups: A (60%), B (10%), C (20%), and D (10%) (Table 2). These haplotypes were defined by seventeen-point polymorphic sites, revealing thirty-one T↔C and thirteen A↔G transitions. It is worth noting that each individual possessed a unique haplotype, indicating the absence of a direct matrilineal relationship among the individuals under analysis.

To classify the mitochondrial haplogroups, we followed the Phylotree hierarchy [16], which begins with the common ancestor R, followed by N, M, and A. Among the individuals with the C16290T polymorphism, six were categorized into haplogroup A, with four of them belonging to the A2 subgroup due to distinct variants. Individual Abu10 was assigned to haplogroup B2s but also exhibited a variant characteristic of B4. Individuals Abu7 and Abu9 were designated as haplogroup C and shared specific variants, while the individual with the C16223T polymorphism was placed in haplogroup D, with individual Abu6 specifically categorized as D4j1a based on another unique variant.

**Figure 2:**
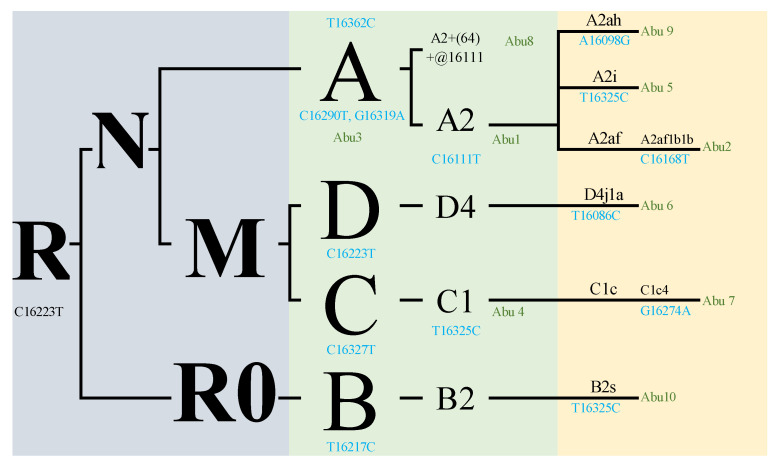
Phylogenetic tree (samples classified in haplogroups and haplotypes). All sorted samples are combined into a resulting tree that includes all rCRS-related polymorphisms. The polymorphisms marked in blue are private polymorphisms for this group, already known by Phylotree. Individuals are marked in green. Based on Phylotree [16], the phylogenetic tree was constructed using the neighbor-joining algorithm. The samples names have been abbreviated for clarity.

The phylogenetic tree (Figure 2) illustrates the presence of various haplogroups and their corresponding samples. In the tree, we observe samples classified under haplogroup A, including A2, A2af1b1b, A, A2i, A2+(64)+ @16111, and A2ah. Additionally, there are samples classified under haplogroup C, namely C and C1c4. The tree also shows a sample categorized under haplogroup D, specifically D4j1a. Lastly, there is a sample denoted as B2s under haplogroup B2. These findings provide valuable insights into the genetic diversity and relationships among the studied population.

**Figure 3:**
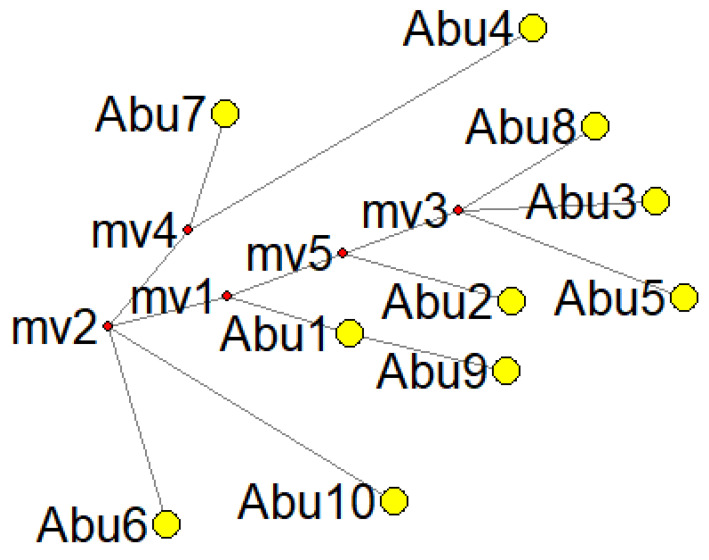
Network analysis of ten HVS-I sequences was performed using the reduced median algorithm. The star contraction option was applied to condense extensive data by identifying phylogenetic clusters into a single ancestral type.

Median-joining (MJ) analysis was employed to explore maternal relationships between individuals in family graves discovered in the Aburrá Valley (Figure 3). The analysis did not reveal direct matrilineal relationships; however, it did show an ancestral connection between an Abu1 node and individual Abu9.

Our study’s results revealed the presence of ten individuals from the Aburrá Valley, representing four founding haplogroups (A, B, C, and D). Each individual exhibited a unique haplotype, indicating the absence of direct matrilineal relationships among them. Haplogroup assignments were made based on specific variants and polymorphisms identified in their sequences.

The haplotype network offers valuable insights into the genealogical relationships among haplotypes. It allows us to assess the degree of divergence between different lineages and estimate the distance to their most recent common ancestor. It is important to note, though, that the haplotype network does not directly establish maternal relationships between specific individuals. Instead, it primarily serves as a tool for understanding the genealogical connections and ancestral origins of different haplotypes. Its relevance lies in elucidating the historical relationships and evolutionary patterns within a population rather than establishing direct relationships between specific individuals.

Furthermore, the results of population diversity in the Aburrá Valley are presented in Table 3, with ‘n’ representing ten individuals and ‘K’ denoting ten sequences analyzed. The nucleotide diversity (π) for the study population is calculated as 0.012. Analysis of the Tajima index (-D) during pre-Hispanic periods indicates population expansion, with a result of −0.72937 and a p-value greater than 0.05; which suggests no significant differences between the analyzed samples [14].

**Figure 4:**
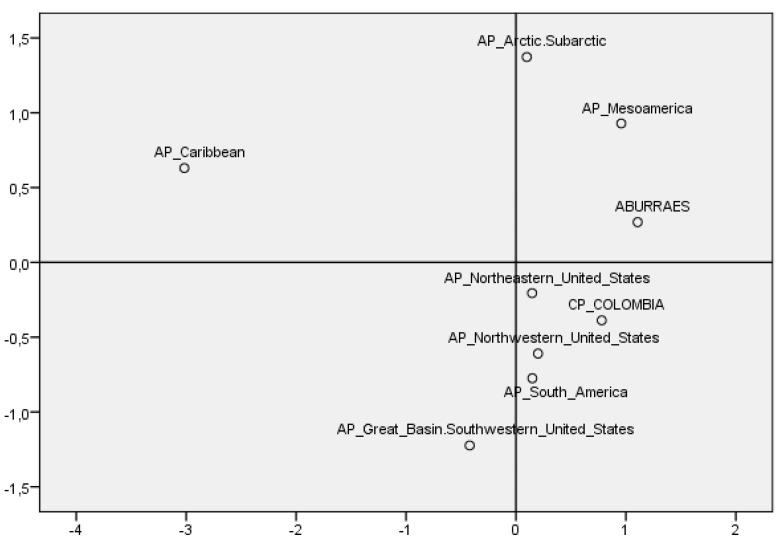
MDS analysis comparing haplotype frequencies of pre-Hispanic populations. AP: ancient population; CP: current population (the stress value for the matrix is 0.05841, with an RSQ of 0.98776).

In line with the research conducted by Raff [15], a multiscale analysis comparing seventy-eight ancient populations across the Americas with twenty contemporary indigenous Colombian populations highlights the highest genetic similarity in the Aburráes population (Figure 4). 

This similarity is particularly striking with pre-Hispanic populations in Mesoamerica, as well as in the Arctic and Sub-Arctic regions. The study reveals the presence of haplogroups A, C, and D in both current and ancient populations throughout the Americas. Notably, haplogroup B is absent in the Caribbean, while haplogroup X is reported to be in low proportions in the northern part of the continent. Haplogroup M, on the other hand, is exclusively identified in an ancient population in the northwestern United States and not in current populations (Table 4).

**Figure 5:**
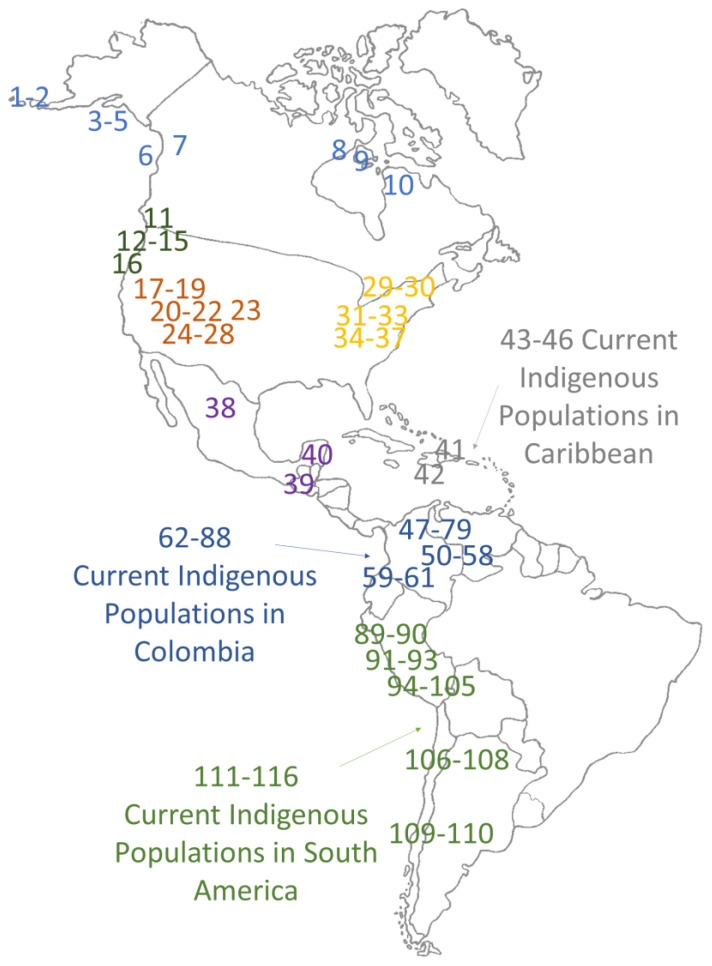
Geographic locations of analyzed populations in the Americas. These zones are color-coded to simplify identification: blue: Arctic and Subarctic; green: northwestern United States; orange: Great Basin; yellow: southwestern United States; purple: Mesoamerican regions; gray: Caribbean; dark blue: Colombia and light green: South America.

The dataset analyzed includes archaeological sites categorized into various geographic zones, namely the Arctic/Subarctic, northwestern United States, Great Basin/southwestern United States, northeastern United States, Mesoamerica, Caribbean, and South America. This regional categorization, as presented in Table 4, is primarily based on the work of Raff [15]. However, it has been refined and expanded upon through recent research, with a particular focus on the inclusion of Colombia. The geographic distribution of these analyzed zones across the Americas is visually represented in Figure 5. Which employs a color scheme for easy identification of geographical locations and highlights the diverse array of regions covered in our study.

For more comprehensive information, including the haplogroup frequencies used for the multidimensional scaling (MDS) analysis, please consult Table 4. The specific matrix of *F_ST_* values can be found in Table S1.

The distribution of mitochondrial haplogroups among the individuals revealed several patterns. Five individuals with the C16290T polymorphism were assigned to haplogroup A, with four of them specifically identified as A2. Notably, individual Abu10 was assigned to haplogroup B2s but also exhibited a variant typical of B4. Meanwhile, Individuals Abu4 and Abu7 were assigned to haplogroup C and shared specific variants. On the other hand, the individual with the C16223T polymorphism was placed in haplogroup D, with individual Abu6 classified as D4j1a, based on another specific variant (Table 5). These findings provide insights into the diversity of mitochondrial haplogroups in the studied population.

## 4. Discussion

The findings of this study offer valuable genetic insights into the population history of pre-Hispanic inhabitants in the Aburrá Valley region. This research primarily focused on the analysis of mitochondrial DNA (mtDNA), which has provided significant information about migratory patterns, interactions, and genetic relationships among ancient American populations.

Studying ancient populations is a multidisciplinary endeavor that combines genetics and archaeology to unravel the complex tapestry of human history. Haplogroups, which are genetic markers passed down through generations, serve as invaluable tools for understanding ancient population dynamics. In this research, we present a comprehensive table featuring archaeological sites across distinct geographic zones, each holding a wealth of genetic information in the form of haplogroups A, B, C, D, X, M, and H.

Variations in haplogroup distribution are evident across these geographic zones, indicating that different regions were inhabited by populations with unique genetic profiles. By examining these archaeological periods, we can observe how haplogroup distributions evolved over millennia; shedding light on genetic adaptations and the ebb and flow of ancient populations. Comparative analysis of haplogroups allows us to reconstruct population movements, migrations, and genetic relationships, providing insights into the interactions between ancient human groups. However, it is crucial to consider sample sizes, as they impact the statistical robustness of genetic findings. Larger sample sizes lead to more reliable insights.

Regarding genetic diversity, the Aburráes population in Colombia exhibited a genetic diversity level of 0.644. When compared to various populations from different geographic regions and archaeological periods, this falls within the range of genetic diversity observed across these populations. Some populations displayed higher genetic-diversity values, while others had lower values. Thus, reflecting the intricate tapestry of human genetic variation across different time periods and geographical locations. It is important to note that, in general, a larger number of samples provide a more accurate reflection of the true diversity of the population or community being studied. Therefore, to obtain reliable and representative results, it is recommended to use a significant number of samples in the diversity analysis.

It is important to emphasize that the haplotype network does not directly establish maternal relationships between specific individuals. Instead, its primary function is to elucidate genealogical connections and ancestral origins among various haplotypes. Its significance lies in unveiling historical relationships and evolutionary patterns within a population rather than confirming direct relationships between specific individuals.

Regarding the Colinita tomb, it’s worth noting that the eight adult individuals found within it are not from the same family. This information holds significant value for archaeologists and anthropologists as it provides insights into burial rituals and helps determine whether the tombs are related to a single family or not.

The meticulous examination of various haplogroups, including A2, B2, C1, and D4j1a, has provided valuable insights into the intricate demographic processes that shaped the region. Notably, Haplogroup A2, renowned for its prevalence in the high latitudes of the Americas and its historical ties to Siberian and Eskimo tribes, has emerged as a pivotal contributor to the initial waves of migration into the continent [16,74]. Moreover, the identification of three distinct sub-haplogroups within A2 among pre-Hispanic Antioquia populations signifies local genetic diversification, underscoring the region’s role as a genetic melting-pot. 

This research delves deeply into the dynamics of these populations. The discovery of A2af1b1b within contemporary populations in Panama and Costa Rica carries profound implications, pointing to significant demographic events approximately ten thousand years ago. While this specific haplogroup is presently observed in only two individuals—one from Chiriqui, Panama, and the other from Costa Rica—it originated 4700 years before the present, with a time to most recent common ancestor (TMRCA) of 1000 years before the present [78].

Most intriguingly, the study identifies the presence of the A2af1b1b sub-haplogroup in an individual from the Valle de Aburra. This finding suggests that A2af1b1b has deep historical roots in the region, indicating its presence within ancient populations. Furthermore, the fact that this haplogroup is still present in contemporary populations emphasizes its enduring legacy [78]. This underscores the notion that A2af1b1b is not just a relic of the past but has persisted through generations and is still part of the genetic makeup of current populations in the region.

The enduring presence of Haplogroup A2ah for over five millennia across various regions offers a compelling narrative of historical population movements. This relatively rare lineage among Native Americans, discerned through comprehensive analyses of complete mitochondrial genomes, is characterized by the uncommon combination of tandem transitions, T16097C and A16098G. Which is rarely encountered in mitochondrial DNA databases [94]. 

Within the larger Haplogroup A2, A2ah emerges as a distinct branch within the Native American mitochondrial family. This research marks a significant milestone in the field of genetics. Notably, for the first time, researchers have unveiled a mitochondrial lineage in the Andean region that was previously believed to be exclusive to the South American lowlands. Specifically, they have identified the A2ah lineage in the Andean area, even within a pre-Hispanic individual. The implications of this discovery are profound, raising intriguing questions about the potential gene flow between the Andean and lowland regions before the Spanish arrival [95].

Archaeological evidence indicates interactions and trade among diverse populations across various eco-regions during pre-Hispanic times. The presence of trade networks, such as those connected to the Quebrada de La Cueva ravine, which housed the Pukara de La Cueva, suggests extensive pre-Hispanic connectivity. Yet, whether these networks also facilitated gene-flow remains uncertain. This underscores the significance of ancient DNA studies in shedding light on this aspect of pre-Hispanic population dynamics [96].

If indeed gene-flow was a regular occurrence between highland and lowland pre-Hispanic populations, it becomes plausible that certain genetic variants from one region could be found in the other. The identification of an infrequent mitochondrial haplotype from the South American lowlands in the Andean area in this study lends support to this hypothesis. To further investigate this proposition, the researchers recommend expanding genetic analyses to encompass other individuals retrieved from Pukara de La Cueva at the population level. Additionally, isotopic analysis could offer insights into the region where the analyzed individual resided during their early years [97].

The research suggests an approximate origin dating back to 5200 years ago, with some within-clade variations. Geographically, this lineage appears to be primarily concentrated in Bolivia and its adjacent regions with a limited distribution [98,99,100].

The discovery of the A2ah mitochondrial lineage in the Andean region, as previously discussed, presents an intriguing parallel to the genetic findings in the Valle de Aburra. In both instances, researchers have uncovered the existence of uncommon mitochondrial haplogroups in regions that defied earlier expectations.

This shared discovery of sub-haplogroup A2ah among both ancient and contemporary Native American populations emphasizes its profound historical importance and its enduring legacy within the tapestry of mitochondrial DNA. The presence of this lineage in diverse temporal contexts underscores its resilience and persistence through time.

By delving deeper into the study of this unique lineage, researchers can unlock invaluable insights into the historical narratives, migration patterns, and genetic tapestry of Native American communities. This connection between seemingly distant regions challenges preconceived notions and beckons for further exploration into the intricate web of human genetic heritage across the Americas.

The genetic narrative of the A2i haplogroup, explored in Achilli et al.’s 2008 study within the Valle de Aburra [75], finds resonance in the genetic tapestry of Alaska. Recent studies involving mitogenomes from Alaska have brought to light a range of haplogroups, including A2, A2a, and A2i. The intriguing presence of A2i in both locales speaks to the remarkable genetic continuity and adaptation across distinct regions and epochs [85,86,87].

Within the complex genetic framework of lineage C1, a thorough network analysis has unveiled a compelling historical narrative. This analysis, which considered sequences from diverse regions such as the Amur River, Japan, and the Americas, lends support to the notion that the foundational haplotype for Native American C1 finds its roots in the Amur River region, echoing previous hypotheses proposed by Starikovskaya [86].

The C1c subclade, an exclusive genetic branch within haplogroup C1 exclusive to Native American populations, stands as a testament to the deep ancestral ties between indigenous peoples and the Americas. C1c plays a pivotal role in genetic and phylogenetic investigations, offering a unique perspective into the evolutionary history and migration patterns of specific groups. This subclade provides invaluable insights into the genetic diversity of Native American populations and their ancestral journeys [87].

The research also delved into broader colonization patterns. The presence of haplogroup B2 across diverse Native American populations adds weight to the idea of an early expansion from North to South America. Additionally, the widespread distribution of haplogroup C1 and its subclades suggests a unified migratory wave that played a crucial role in populating the Americas. The identification of variant 16298C in Abu4 within haplogroup C underscores the distinctive genetic legacy of pre-Hispanic Native American settlers. 

Moreover, the presence of Haplogroup C1c, with its older coalescent age and evidence of intra-haplogroup variation, in both Mexican Americans and ancient Valle de Aburra populations hints at a captivating connection. This shared genetic heritage suggests that the genetic diversity within Haplogroup C1c, as well as its sub-branches, was firmly established among ancestral populations before their migration and expansion into the Americas. The exploration of this haplogroup’s distribution and variation across populations promises profound insights into historical movements and genetic relationships among these groups, ultimately unraveling the intricate history of Native American origins and migrations.

Within the realm of Native American haplogroups, B2 emerges as a distinct lineage exclusive to indigenous populations of the Americas. Setting it apart from the other major founding haplogroups (A2, C1, D1, and X2a), the basal B2 lineage lacks defining variants in the control region (CR). This unique feature presents a challenge in distinguishing B2 from closely related haplogroups, a critical task for distinguishing Native American from Asian haplotypes [91].

Compelling evidence suggests that the initial wave of people entering what is now Mexican territory carried with them the A2 and B2 haplogroups around 15–18-thousand years ago. The distribution of individuals with higher frequencies of B2 or C1 compared to A2 indicates a specific migration pattern. A2, B2, and C1 appear to have been primarily dispersed along a Pacific coast route, spanning approximately 800 km along the Neovolcanic axis region. Subsequently, they made their way southward along the Pacific coast route, eventually reaching the Yucatan Peninsula via the Gulf coast route.

It is noteworthy that the Neovolcanic axis region, characterized by the presence of significant rivers like Lerma and Balsas, acts as a natural barrier against adverse Pacific weather conditions. This geographical feature likely played a pivotal role in facilitating the spread of these haplogroups [86,91].

Sub-haplotype D4j1a stands out from the norm when it comes to substitutions observed in mitochondrial DNA clusters associated with the southern distribution of aboriginal Siberian peoples. These substitutions are typically linked to major mitochondrial DNA haplogroups prevalent in East and South Asia. The intriguing presence of sub-haplotype D4j1a among both aboriginal Siberian populations and the ancient inhabitants of the Valle de Aburra unveils a fascinating genetic connection spanning vast geographical distances. Importantly, this sub-haplotype diverges from the usual mitochondrial DNA clusters found in aboriginal Siberian populations, aligning instead with major mitochondrial DNA haplogroups prevalent in East and South Asia [87,88,89,90,91,92,93].

The genetic connection between Siberia and the Valle de Aburra holds the potential to illuminate ancient migration patterns and the interactions between diverse populations. To gain a deeper understanding of the historical context and the implications of this shared genetic marker, further genetic and archaeological investigations are imperative (Figure 5).

Understanding the genetic connection between Siberia and Valle de Aburra offers a glimpse into the intricate journey of ancient human migrations. It suggests that populations from distant regions might have come into contact, possibly through migratory movements, trade routes, or cultural exchanges.

To provide a meaningful context for this genetic link, it is crucial to highlight the fundamental role of archaeological investigations, which, in combination with genetics, offer a more accurate representation of ancient life. The genetic relationship between the Valle de Aburra and Siberia, along with its proximity to Mesoamerica, suggests the diffusion of genetic markers across populations. Mesoamerica’s genetic diversity, a result of its complex history, can provide insights into ancient cultural exchanges and trade routes. A multidisciplinary approach, encompassing genetics and archaeological excavations, is essential for a comprehensive understanding of the history and implications of this shared genetic marker.

The revelation of a genetic connection weaving through Siberia, Mesoamerica, and Valle de Aburra is nothing short of enthralling. This revelation acts as a beckoning call, compelling us to embark on an expedition of profound historical significance. Expanding our investigation into Mesoamerica opens doors to unraveling ancient ties, migratory routes, and the intricate web of interactions that wove together diverse populations. In the realm of collaborative genetic and archaeological exploration, we stand on the precipice of unearthing the narratives of our forebears, shedding brilliant light upon the enigmatic pathways of our shared past.

Comparisons with other populations across the Americas revealed that the Aburráes population showed the highest genetic similarity with pre-Hispanic populations in America. The results presented in our study contribute to the understanding of the genetic diversity and ancestral connections of the Aburráes population. These findings have implications for the population history and migration patterns in the Americas.

Furthermore, the study sheds light on population relationships across regions. The genetic affinity between the Aburráes and Mesoamerican and Arctic–sub-Arctic populations showcase intricate gene flow and migratory dynamics. These findings align with the hypothesis of a coastal migration route and potential interactions between the indigenous Chibchas population and the Mayan population.

The primary limitation of this study arises from its exclusive focus on the HVS-I of ancient mtDNA. This choice was necessitated by the inherent challenges in working with degraded and ancient DNA samples. While the results obtained are indeed intriguing and informative, it is imperative to recognize and acknowledge this limitation.

Likewise, it is advisable to approach the study’s conclusions with a degree of caution. This caution is particularly warranted due to the relatively small sample size, consisting of only 10 individuals, and the restriction of the analysis to a single mitochondrial region (HVS-I).

Despite these challenges, it is noteworthy that the study was successful in elucidating important haplogroups. These findings make a significant contribution to our understanding of the genetic history of the ancient population under investigation.

## 5. Conclusions

In conclusion, the present study has provided valuable insights into the genetic diversity and population history of pre-Hispanic inhabitants in the Aburrá Valley region. The identification of various mitochondrial haplogroups, particularly A2 and its sub-lineages (A2af1b1b, A2ah, and A2i), Haplogroup B2, Haplogroup C1, and Haplogroup D4j1a, has shed light on the complex migratory patterns and interactions of ancient populations in the Americas.

One of the most striking findings of this research is the presence of distinct sub-haplogroups within the larger haplogroups, such as A2af1b1b, A2ah, and A2i. These sub-haplogroups not only reveal local genetic diversification but also highlight the enduring legacy of certain genetic lineages in the region.

While the genetic data presented in this study contribute valuable insights, it represents only a starting point in the investigation of the region’s population history. The pursuit of more extensive and refined research will undoubtedly bring us closer to a deeper appreciation of the rich and complex human history that unfolded in the Aburrá Valley throughout the ages.

Ongoing research efforts are vital to fully unravel the region’s population history. By continuously exploring and integrating diverse data sources, we can achieve a more nuanced understanding of the region’s past, its connections to broader human migrations in the Americas, and the factors shaping its distinctive genetic landscape.

## Figures and Tables

**Table 1:** Notable Colombian archaeological sites dated between the late Pleistocene and early Holocene.

Place	Region	Dates BP Not Calibrated
Tocaima-Cundinamarca	Pubenza	16,400 ± 420
Zipaquirá-Cundinamarca	El Abra	12,400 ± 160
Tocancipá-Cundinamarca	Tibitó 1	11,740 ± 110
Tequerdama-Cundinamarca	Tequendama 1 y 2	9740 ± 135
Nemocón-Cundinamarca	Checua	8500–3000
Roncesvalles -Tolima	Jordan	12.910 ± 60–9760 ± 160
Porce 045-Antioquia	Porce	7.080 ± 130
La Morena Envigado-Antioquia	Porce River High Basin	10,060 ± 60–9680 ± 60
Porce, Primavera II-Antioquia	Porce River Low Basin	7730 ± 170
Medellín Guayabal-Antioquia	La Colinita	880 ± 30
Medellín, Robledo-Antioquia	Cerro El Volador T10	950 ± 70
Medellín, Robledo-Antioquia	Cerro El Volador T5	530 ± 80
Medellín, Robledo-Antioquia	Cerro El Volador T13	480 ± 60
Medellín, Robledo-Antioquia	Cerro El Volador T9	420 ± 50
Medellín, Robledo-Antioquia	Cerro El Volador T8	330 ± 60
Envigado, Loma del Barro-Antioquia	Alto de las Flores	140 ± 30

**Table 2:** Results of haplogroups and haplotypes identification based on the HVS-I sequence for each of the analyzed samples with the gender assignment, the origin of the excavation site and type of tissue used for DNA extraction.

Sample	Gender	Site	Description	Range	Haplogroup	Quality	Polymorphisms
Abu 1	Indet.	La Colinita	Molar	16,024–16,569	A, A2	86%	16111T 16223T 16290T
Abu 2	Indet.	La Colinita	Molar	16,024–16,569; 1–576	A, A2af1b1b	60%	16168T 16223T 16290T 16362C
Abu 3	Indet.	La Colinita	Molar	16,024–16,569	A	94%	16129A 16223T 16290T 16319A 16362C
Abu 4	Indet.	La Colinita	Molar	16,024–16,569	C	97%	16223T 16288C 16298C 16311C 16327T
Abu 5	Indet.	La Colinita	Molar	16,024–16,569	A, A2i	100%	16111T 16223T 16290T 16319A 16325C 16362C
Abu 6	Indet.	La Colinita	Molar	16,024–16,569; 1–576	D, D4j1a	65%	16086C 16183C 16223T
Abu 7	Indet.	La Colinita	Molar	16,024–16,569;	C, C1c4	94%	16223T 16274A 16327Y
Abu 8	Indet.	La Colinita	Molar	16,024–16,569	A, A2+(64)+ @16111	82%	16290T 16319A 16362C
Abu 9	Female	Cerro el Volador	Bone remains	16,024–16,569; 1–576	A, A2ah	81%	16098G 16111T 16223T 16290T
Abu 10	Indet.	Alto de las Flores	Bone remains	16,024–16,569; 1–576	B2, B2s	62%	16217C 16325C

**Table 3:** Measures of diversity in the Aburráes population from the mitochondrial sequences of the HVS-I. Where k number of different sequences; H, haplotype/genetic diversity; π, nucleotide diversity and Tajima’s index (-D) and *p* value.

Population	K	H	π	Tajima’s	*p*-Value
Aburráes	10	0.644	0.012	−0.72937	0.254

**Table 4:** Table of haplogroups, showing genetic diversity across ancient and contemporary populations throughout the American continent. The genetic diversity (h) for various archaeological sites in different geographic zones, including the Aburráes region in Colombia. Sites with “N.C.” values indicate that there is no genetic diversity data available for those sites. AP = 0 for modern indigenous populations.

Position on the Map	Geographic Zone	Archaeological Site	AP	N	A	B	C	D	X	M	H	Reference
1	Arctic, Subarctic	Early Aleuts	3500–1200	11	8	0	0	3	0	0	0.436	[17]
2	Arctic, Subarctic	Late Pre-Contact Aleuts	1000–400	52	12	0	0	40	0	0	0.362	[17]
3	Arctic, Subarctic	Brooks River	938–1318	8	5	2	0	1	0	0	0.607	[18]
4	Arctic, Subarctic	Hot Springs	2070	3	1	0	0	2	0	0	N.C.	[18]
5	Arctic, Subarctic	On-Your-Knees Cave	10,300	1	0	0	0	1	0	0	N.C.	[19]
6	Arctic, Subarctic	Mink Island	1029–1215	6	1	0	0	5	0	0	0.333	[18]
7	Arctic, Subarctic	Tatshenshini-Alsek Glacier	550	1	1	0	0	0	0	0	N.C.	[20]
8	Arctic, Subarctic	Thule	1130–628	15	15	0	0	0	0	0	0	[21]
9	Arctic, Subarctic	Sadlermiut	977–682	18	10	0	0	8	0	0	0.523	[21]
10	Arctic, Subarctic	Dorset	2260–1216	2	0	0	0	2	0	0	N.C.	[21]
11	Northwest United States	China Lake and Big Bar Lake	4975	3	1	0	0	0	0	2	N.C.	[19]
12	Northwest United States	Plateau Salish	200	11	3	6	1	1	0	0	0.673	[19]
13	Northwest United States	Plateau Sahaptian	200	8	0	4	2	2	0	0	0.714	[19]
14	Northwest United States	Wishram	200	33	7	17	0	9	0	0	0.635	[19]
15	Northwest United States	Vantage	500–1500	7	2	0	1	3	1	0	0.81	[19]
16	Northwest United States	Paisley 5 Mile Point Caves	14,000	2	1	1	0	0	0	0	N.C.	[22]
17	Great Basin, Southwest United States	Cecil	3600–2860	16	0	1	9	6	0	0	0.575	[23]
18	Great Basin, Southwest United States	Cook	2000	23	1	2	10	10	0	0	0.64	[23]
19	Great Basin, Southwest United States	Applegate	1765–2055	6	0	2	4	0	0	0	0.533	[23]
20	Great Basin, Southwest United States	Pyramid Lake	860–5905	18	2	6	0	10	0	0	0.601	[24]
21	Great Basin, Southwest United States	Pyramid Lake (Wizards Beach)	9200	1	0	0	1	0	0	0	N.C.	[24]
22	Great Basin, Southwest United States	Stillwater Marsh	290–3290	21	1	8	0	12	0	0	0.552	[24]
23	Great Basin, Southwest United States	Hourglass Cave	8000	1	0	1	0	0	0	0	N.C.	[25]
24	Great Basin, Southwest United States	Tommy Site	850–1150	36	1	25	5	5	0	0	0.492	[26]
25	Great Basin, Southwest United States	Mine Canyon	650–850	12	7	4	1	0	0	0	0.591	[26]
26	Great Basin, Southwest United States	Fremont	500–1500	30	0	24	4	2	0	0	0.349	[27]
27	Great Basin, Southwest United States	Anasazi	1010–2010	38	4	27	7	0	0	0	0.462	[27]
28	Great Basin, Southwest United States	Western Basketmaker II	2500–1300	23	3	18	1	1	0	0	0.383	[27,28]
29	Northeast United States	Great Western Park	800	6	2	0	4	0	0	0	0.533	[29]
30	Northeast United States	Glacial Kame	2900	18	3	11	3	1	0	0	0.601	[29]
31	Northeast United States	Morse	2700	9	1	3	5	0	0	0	0.639	[29]
32	Northeast United States	Orendorf	800	11	5	0	3	3	0	0	0.709	[29]
33	Northeast United States	Norris Farms	700	108	34	13	46	9	6	0	0.702	[25]
34	Northeast United States	Pete Klunk Mound Group	1825	39	9	5	19	5	1	0	0.694	[30]
35	Northeast United States	Schild Mississippian	900	47	18	6	11	4	8	0	0.762	[31]
36	Northeast United States	Schild Late Woodland	1200	19	5	1	4	1	8	0	0.743	[31]
37	Northeast United States	Ohio Hopewell Mound Group	1700	34	14	3	10	7	0	0	0.715	[32]
38	Mesoamerica	Tlatelolco Post-Classic Aztec	500–675	23	15	3	1	4	0	0	0.574	[33]
39	Mesoamerica	Maya (Xcaret)	480–1400	24	20	1	2	0	0	0	0.236	[34]
40	Mesoamerica	Maya (Copan)	750–1300	9	0	0	8	1	0	0	0.222	[35]
41	Caribbean	La Caleta (Tainos)	1330–320	24	0	0	18	6	0	0	0.391	[36]
42	Caribbean	Cuba (Ciboneys)	4700–1620	15	1	0	9	5	0	0	0.562	[37]
43	Caribbean	Boruca	0	14	3	10	0	1	0	0	0.473	[38]
44	Caribbean	Huetar	0	27	19	1	0	7	0	0	0.453	[39]
45	Caribbean	Ngöbe	0	46	31	15	0	0	0	0	0.449	[40]
46	Caribbean	Tainos	0	19	0	0	15	4	0	0	0.351	[41]
47	Colombia	La Purnia (Guane)	1090	17	6	7	0	4	0	0	0.875	[42]
48	Colombia	Jerico (Lache)	700	5	2	3	0	0	0	0	0.600	[43]
49	Colombia	Andes Orientales Colombianos (Muisca, Lache, Guane, Checua, Aguazuque)	1200–700	13	1	7	4	1	0	0	0.654	[44]
50	Colombia	Madrid (Muisca)	2000	6	0	6	0	0	0	0	0	[45]
51	Colombia	Candelaria (muisca)	900	10	10	0	0	0	0	0	0	[46]
52	Colombia	Antioquia (Aburraes)	800	10	6	1	2	1	0	0	0.644	[43]
53	Colombia	Malambo	3000	6	0	6	0	0	0	0	0	[47]
54	Colombia	Sogamoso (Muisca)	800–1500	13	9	3	1	0	0	0	0.500	[48]
55	Colombia	Nemocon (Checua)	8000	5	3	1	1	0	0	0	0.700	[3]
56	Colombia	Herrera (Muisca)	1400–100	7	7	0	0	0	0	0	0	[49]
57	Colombia	Tibanica (Muisca)	800	18	12	2	0	4	0	0	0.523	[50]
58	Colombia	Agroalfarero (early Muisca)	900	24	13	6	4	1	0	0	0.641	[51]
59	Colombia	Páez 2	0	36	10	3	10	12	1	0	0.747	[52]
60	Colombia	Valle del Cauca (Calima)	800–1200	17	5	8	2	2	0	0	0.706	[43]
61	Colombia	Ovando (Quimbaya)	1500–700	7	0	6	0	1	0	0	0.286	[53]
62	Colombia	Desano	0	20	3	3	9	5	0	0	0.726	[52]
63	Colombia	Curripaco	0	22	1	9	8	3	1	0	0.710	[52]
64	Colombia	Wayuu	0	30	8	8	13	0	1	0	0.692	[12,54]
65	Colombia	Guane	0	17	6	7	0	4	0	0	0.691	[42]
66	Colombia	Waunana	0	40	2	19	11	7	1	0	0.682	[55]
67	Colombia	Ingano	0	48	19	17	11	1	0	0	0.679	[52]
68	Colombia	Tule-Cuna	0	30	15	8	6	0	1	0	0.660	[55,56]
69	Colombia	Tucano	0	14	1	3	1	8	1	0	0.659	[52]
70	Colombia	Embera	0	21	2	11	6	2	0	0	0.657	[52]
71	Colombia	Antioquia	0	38	18	10	0	10	0	0	0.654	[57]
72	Colombia	Maya	0	27	14	6	4	2	0	0	0.652	[56]
73	Colombia	Embera	0	21	7	10	1	2	0	0	0.647	[55]
74	Colombia	Tatuyo	0	10	4	0	5	1	0	0	0.644	[52]
75	Colombia	Zenu	0	34	5	11	17	1	0	0	0.642	[55]
76	Colombia	Chibcha	0	15	6	2	4	2	1	0	0.781	[52]
77	Colombia	Puinave	0	61	5	31	20	4	1	0	0.633	[52]
78	Colombia	Cauca	0	60	11	5	38	5	1	0	0.560	[57]
79	Colombia	Páez 1	0	31	18	2	11	0	0	0	0.551	[55]
80	Colombia	Guane-Butar	0	33	4	21	0	8	0	0	0.538	[55]
81	Colombia	Cubeo	0	24	8	2	7	6	1	0	0.764	[52]
82	Colombia	Guambiano	0	80	5	6	59	10	0	0	0.436	[57]
83	Colombia	Arsario	0	28	20	0	8	0	0	0	0.423	[12]
84	Colombia	Kuna	0	63	45	18	0	0	0	0	0.415	[12]
85	Colombia	Kogui	0	21	17	0	4	0	0	0	0.324	[12,54]
86	Colombia	Arhuaco	0	21	19	1	1	0	0	0	0.186	[52]
87	Colombia	Ijka	0	31	28	1	2	0	0	0	0.185	[12]
88	Colombia	Chimila	0	56	50	1	1	2	1	0	0.174	[52]
89	South America	North Peruvian Coast	1000	21	6	7	1	7	0	0	0.729	[58]
90	South America	Peruvian Highlanders	550–450	35	3	23	8	1	0	0	0.523	[59]
91	South America	Conchapata	1400–1200	14	4	7	2	1	0	0	0.692	[60]
92	South America	Cuzco	1020–830	1	0	0	0	1	0	0	N.C.	[61]
93	South America	Huari	900–600	18	3	4	10	1	0	0	0.647	[62]
94	South America	Paracas (Peninsula)	2800–2200	10	0	0	3	7	0	0	0.467	[63]
95	South America	Chen Chen	1215–1000	23	9	9	4	1	0	0	0.692	[64]
96	South America	Paracas (Palpa)	2800–2200	28	2	0	4	22	0	0	0.370	[63]
97	South America	Nasca-Rural (Palpa)	2200–1400	37	1	4	8	24	0	0	0.535	[63]
98	South America	Nasca-Urban (Palpa)	2200–1400	28	0	5	12	11	0	0	0.653	[63]
99	South America	Middle Horizon (Palpa)	1400–1000	11	0	3	4	4	0	0	0.727	[63]
100	South America	Pacapaccari (Highlands)	820	16	0	11	5	0	0	0	0.458	[63]
101	South America	Yacotogia	1187	25	1	14	10	0	0	0	0.547	[63]
102	South America	Ocoro	1400–1000	5	1	3	1	0	0	0	0.700	[63]
103	South America	Botigiriayocc	1000–600	12	0	8	4	0	0	0	0.485	[63]
104	South America	Huayuncalla	978	5	1	2	2	0	0	0	0.800	[63]
105	South America	Layuni	1400–1000	9	0	5	3	1	0	0	0.639	[63]
106	South America	North Chile Late Archaic	6000–3900	14	7	5	1	1	0	0	0.659	[65]
107	South America	North Chile Middle Horizon	1650–1000	19	6	8	5	0	0	0	0.690	[65]
108	South America	North Chile Late Intermediate	1000–500	15	3	8	3	1	0	0	0.676	[65]
109	South America	Pampa Grande	1600–1350	19	2	9	0	8	0	0	0.620	[66]
110	South America	Baño nuevo, Tagua Tagua, Camarones	9000–7500	30	9	10	9	2	0	0	0.729	[67]
111	South America	Mapuche	0	39	6	15	8	10	0	0	0.740	[68]
112	South America	Yungay	0	20	1	10	5	4	0	0	0.679	[69]
113	South America	Quechua	0	23	2	13	7	1	0	0	0.605	[70]
114	South America	Yanomami	0	53	0	5	31	15	3	0	0.593	[71]
115	South America	Zoro	0	29	6	0	4	18	1	0	0.571	[72]
116	South America	Xavante	0	24	4	20	0	0	0	0	0.290	[72]

**Table 5:** Distribution of mitochondrial haplogroups in the sampled population. Offering an insightful breakdown of the genetic haplogroups assigned to each individual in the study. This comprehensive presentation aids in the categorization and enhances our understanding of the genetic diversity within the sampled population.

Sample ID	Locality	Haplogroup	Haplotype	Haplotype Global Distribution
Abu 1	La Colinita	A2	16111T 16223T 16290T	Found across the entire continent of the Americas [73]
Abu 2	La Colinita	A2af1b1b	16168T 16223T 16290T 16362C	Specifically present in Panama and Costa Rica [74,75]
Abu 3	La Colinita	A	16129A 16223T 16290T 16319A 16362C	Found across the entire continent of the Americas [73]
Abu 4	La Colinita	C	16223T 16288C 16298C 16311C 16327T	Prevalent in most parts of the American continent [73]
Abu 5	La Colinita	A2i	16111T 16223T 16290T 16319A 16325C 16362C	Concentrated in central North America, including Nebraska, Minnesota, and South Dakota [73], found in ancient populations in Canada [76,77,78]
Abu 6	La Colinita	D4j1a	16086C 16183C 16223T	Found in a small number of individuals in Thailand and Tibet [73,79,80,81,82,83,84,85]
Abu 7	La Colinita	C1c4	16223T 16274A 16327Y	Located in the southern region of Mexico [86,87]
Abu 8	La Colinita	A2	16290T 16319A 16362C	Found across the entire continent of the Americas [73]
Abu 9	Cerro el Volador	A2ah	16098G 16111T 16223T 16290T	Present in North America but more concentrated in South America [73,88,89,90,91]
Abu 10	Alto de las Flores	B2s	16217C 16325C	Specifically found in North America [73,92,93]

## Data Availability

The data supporting the reported results can be found in GenBank flat file: LOCUS OR478541 483 bp DNA linear PRI 17-OCT-2023 DEFINITION Homo sapiens isolate M1-P1_H15995_P1 D-loop, partial sequence; mitochondrial.

## Supplementary Materials

The following supporting information can be downloaded at: https://www.mdpi.com/article/10.3390/genes14112036/s1, Table S1: Genetic Distance Matrix Using Frequency Data from Seven Prehispanic Populations.
